# Criteria-based audit to improve quality of care of foetal distress: standardising obstetric care at a national referral hospital in a low resource setting, Tanzania

**DOI:** 10.1186/s12884-016-1137-z

**Published:** 2016-11-08

**Authors:** Andrew H. Mgaya, Helena Litorp, Hussein L. Kidanto, Lennarth Nyström, Birgitta Essén

**Affiliations:** 1 Department of Obstetrics and Gynaecology, Muhimbili National Hospital, Dar es Salaam, Tanzania; 2 Department of Women’s and Children’s Health/International Maternal and Child Health, Uppsala University, Uppsala, Sweden; 3 Reproductive and Child Health section, Ministry of Health, Community Development, Gender, Elderly and Children, Dar es Salaam, Tanzania; 4 Department of Public Health and Clinical Medicine, Epidemiology and Global Health, Umeå University, Umeå, Sweden

**Keywords:** Caesarean section, Criteria-based audit, Foetal distress, Fetal Doppler, Low-resource setting

## Abstract

**Background:**

In Tanzania, substandard intrapartum management of foetal distress contributes to a third of perinatal deaths, and the majority are term deliveries. We conducted a criteria-based audit with feedback to determine whether standards of diagnosis and management of foetal distress would be improved in a low-resource setting.

**Methods:**

During 2013–2015, a criteria-based audit was performed at the national referral hospital in Dar es Salaam. Case files of deliveries with a diagnosis of foetal distress were identified and audited. Two registered nurses under supervision of a nurse midwife, a specialist obstetrician and a consultant obstetrician, reviewed the case files. Criteria for standard diagnosis and management of foetal distress were developed based on international and national guidelines, and literature reviews, and then, stepwise applied, in an audit cycle. During the baseline audit, substandard care was identified, and recommendations for improvement of care were proposed and implemented. The effect of the implementations was assessed by the differences in percentage of standard diagnosis and management between the baseline and re-audit, using Chi-square test or Fisher’s exact test, when appropriate.

**Results:**

In the baseline audit and re-audit, 248 and 251 deliveries with a diagnosis of foetal distress were identified and audited, respectively. The standard of diagnosis increased significantly from 52 to 68 % (*p* < 0.001). Standards of management improved tenfold from 0.8 to 8.8 % (*p* < 0.001). Improved foetal heartbeat monitoring using a Fetal Doppler was the major improvement in diagnoses, while change of position of the mother and reduced time interval from decision to perform caesarean section to delivery were the major improvements in management (all *p* < 0.001). Percentage of cases with substandard diagnosis and management was significantly reduced in both referred public and non-referred private patients (all *p* ≤ 0.01) but not in non-referred public and referred private patients.

**Conclusion:**

The criteria-based audit was able to detect substandard diagnosis and management of foetal distress and improved care using feedback and available resources.

## Background

In Tanzania, substandard care contributes up to a third of perinatal deaths due to foetal distress that, in the majority of cases, is associated with intrapartum asphyxia of term deliveries [1]. The Tanzania Demographic Health Survey (2010) found a perinatal mortality rate of 36/1000 live births: higher in urban (48/1000 live births) than rural areas (33/1000 live births). In rural Tanzania, birth asphyxia was associated with more than half of neonatal deaths of term deliveries of babies with normal birth weight [2]. At the urban national referral hospital in Dar es Salaam, poor foetal heart monitoring indirectly caused over 40 % of the perinatal deaths in 2007 [3]. However, clinical routines were then improved and perinatal death audits have become a standard procedure since 2009. A recent study at the same hospital [4] showed that the caesarean section (CS) rate increased from 19 to 50 % without significant reduction of perinatal mortality. In the same setting, a clinical audit of indications of CS demonstrated that foetal distress was the second most common indication for CS, while at the same time, it contributed up to one-third of the substandard indications of CS [5]. Thus, improved standards of care in the diagnosis and management of foetal distress depends on local strategic action, including adequate monitoring of pregnancies, and timely evidence-based interventions during delivery [6, 7].

In addition to limited availability of medical technology [8, 9] and a lack of skills in care among providers [1, 7] in the diagnosis and management of foetal distress, a previous interview study [10] of care providers at the national referral hospital showed a pronounced fear and blame culture amongst the staff during an audit and case review to address the issues of poor outcomes. Poor audit-feedback [10], fear of medical litigation [11, 12], and mothers’ and care providers’ desire to deliver a healthy baby [13] can evoke anxiety, resulting in defensive medical practice, and hence substandard decisions related to the performing of CS [14]. As discussed by McGivern and Fischer [15] and Espeland and Sauder [16], reactivity mechanisms associated with care providers’ anxiety come as a result of a shift in the care providers’ focus, from clients’ safety and good care, to professional security, and thus favouring their own good assessment over good client outcomes.

The feasibility of criteria-based audit (CBA) in improving care in low-resource settings has been studied in Ghana and Jamaica [17]. Aligning with the principles of CBA [18], success in quality of care assessment relies on accurate identification of the criteria for standard practice and appropriate case definitions, where a group reflection of consensual standards is preferable compared to individual or universally defined best practice [19, 20]. Additionally, the success of implementations of audit feedback interventions should take into account the accessibility of resources [21], and depends on the leadership and involvement of care providers [22]. The aim of this study was to test whether a CBA could improve the diagnosis and management of foetal distress within the current context of a national referral hospital in Tanzania.

## Methods

### Study design, period and the study population

The baseline CBA was conducted from October 2013 to April 2014. After implementing the recommended changes, the re-audit was performed from July to November 2015 using case files of delivered patients with a physician diagnosis of foetal distress. The inclusion criterion was delivery of a single, term foetus in cephalic presentation. The exclusion criteria included women that had obstetric or medical condition that could have presented as, or led to, foetal distress; thus biased the evaluation of diagnosis, management or outcome of foetal distress. The cases include premature membrane rupture and severe medical conditions such as malaria, eclampsia, cardiac disease, severe anaemia, as defined as haemoglobin of <7 g/dl in the national maternal and child heath guideline [23], and obstructed labour (baseline audit *n* = 82 and re-audit *n* = 76). On a weekly or 2-weekly basis, a member of the audit team performed a case validation check by comparing details of randomly selected completed audit forms and case files retrieved from medical records, using patient registration number and names.

### Study setting

The CBA was performed at Muhimbili National referral Hospital (MNH) which serves 4.4 million inhabitants of Dar es Salaam city (*National Census*, 2012) and the neighbouring Pwani region. The obstetric population consists of patients referred from the three regional hospitals in Dar es Salaam (Mwananyamala, Amana and Temeke), the two rural district hospitals (Mkuranga and Bagamoyo hospital) in the Pwani region, and self-referred patients. From 2013 to 2015, the average maternal mortality rate was 301/100,000 live births, the stillbirth rate was 78/1000 live births, and the neonatal distress rate (Apgar score 1–6 at the 5^th^ minute after birth) was 54/1000 live births, of women delivered at MNH. The MNH delivers 8,000–10,000 women annually, and receives both public non-paying and private paying patients. Private patients are managed in a similar way to those in the public patients, except that they receive additional services, such as comfortable accommodation, and the privilege of being attended by a specialist of their choice. There are three shifts for nurses working in the labour ward, each with six midwives. One consultant, one specialist and two residents are on call every day. The hospital has only two obstetric theatres and one functioning vacuum extractor. The standard foetal monitoring technique is Pinard auscultation and Fetal Doppler. Cardiotocography (CTG) and scalp blood sample for blood gas/pH analysis is not available.

Information from antenatal care cards and medical records is routinely entered in the maternity register and, since 1998, this information has been computerised. The obstetric database constitutes the main source of obstetric data for staff’s research and audits [24].

### Development of the audit form and audit procedure

The audit form was developed to capture patients’ background data and clearly defined indicators of the process of diagnosis and management of foetal distress. A panel of experts, a statistician and two obstetricians, with experience in clinical audit reviewed the audit form for clarity and relevance in identifying appropriate measure of process and outcome in care of foetal distress. During the review process, inappropriate items were discussed and either removed or modified. The form was piloted for 30 patients, and continuous revision was performed to the satisfaction of the experts and data collectors.

**Fig. 1:**
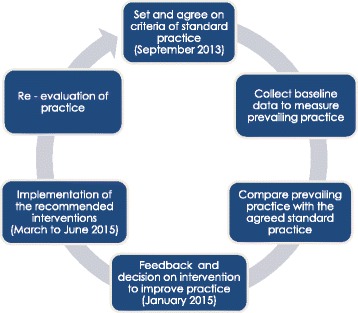
Criteria-based audit cycle

The clinical audit was performed using the criteria-based audit cycle (Fig. 1). A clinical audit can be defined as systematic and critical analyses of the “quality of medical care, including the procedures used for diagnosis and treatment, the use of resources and the resulting outcome and the quality of life of the patient” [25].

#### Set and agree on criteria of standard practice (first step)

The criteria were developed after scrutinising the guidelines from the World Health Organization [26] and International Federation of Gynaecologist and Obstetrician (FIGO) [9], National guidelines [27], and peer-reviewed scientific publications, to extract relevant standards for diagnosis and management of foetal distress that were further reviewed by four obstetricians, two midwives and eight obstetric residents. The reviewed list of criteria was later authenticated in a departmental meeting that included 55 staff from the maternity ward, namely, doctors, midwives, an anaesthesiologist, a pharmacist, a laboratory technician and ward attendants. In order to optimally operationalise the guidelines, care providers agreed to term “foetal heart late decelerations” abnormalities as “irregular foetal heart rate”, which carried a common meaning of “persistent abnormal foetal heartbeats between the uterine contractions” by all healthcare professionals, regardless of their skills and expertise [28]. The decision of the final criteria of standard diagnosis and management was reached by consensus. In case there was no consensus, the most senior in the group made the final decision. The final agreed list of criteria for the diagnosis and management of foetal distress is presented in Tables 1 and 2.

**Table 1:** List of standards for diagnosis of foetal distress

Fulfilment of standards for diagnosis should include one major and one minor criterion.	
Major criteria:	
1.	Irregular foetal heartbeats (non-uniform foetal heart rate between the uterine contractions)
2.	Abnormal foetal heart rate (>180 or <100 beats/min)
Minor criteria:	
1.	Persistence of irregular heartbeats despite hydration and change of maternal position
2.	Fresh meconium-stained liquor
3.	Reduced foetal movement

**Table 2:** List of standard for management of foetal distress

Fulfilment of criteria for management should include ALL of the following:
Standard management guidelines
1. Intravenous rehydration (≥1 l of crystalloids)
2. Repositioning of the mother to lateral lying position
3. Review by a senior specialist (at least once during the process of labour to delivery, either by him/herself, by phone or during major/service ward round)
Standard preoperative management
1. Drained urinary bladder (with indwelling urethral catheter)
2. Blood-typing and cross-matching
3. Administration of antibiotics (broad spectrum)
4. Sought patient’s informed consent
5. Pre-operation checklist used (verify the pre-operative protocol and timelines of intervention from decision to arrival in theatre)
6. Caesarean section should commence ≤1 h after decision (Decision to theatre arrival interval ≤30 min and theatre arrival to delivery interval ≤30 min)

#### Collect baseline data to measure prevailing practice (second step)

The aim of this step was to evaluate the prevailing practice that was later compared with the agreed standards. Two trained data collectors, registered nurses in the post-natal obstetric ward, checked the delivery book every morning and every afternoon and, identified deliveries with diagnosis of foetal distress and then traced case files using patients’ names and file numbers. The case files included clinical notes, antenatal cards and partographs. Data such as age, parity, patient referral category, mode of delivery, details of foetal monitoring, state of liquor and interventions following diagnosis, were collected using a pre-tested audit forms. The filled audit forms were reviewed for completeness before storage. In case of missing information in the case file, other sources of information, including the post-natal ward admission and report book, theatre analysis book and interview from patients, were used to fill in the missing details. Data quality control measures included comparison of randomly selected filled audit forms to the respective details in the case files, and frequencies of each variable were checked to determine missing data.

The prevailing practise was measured according to the agreed standard diagnosis and management of foetal distress. Therefore, substandard diagnosis was a physician documented diagnosis of foetal distress that did not fulfil at least one major and one more criteria. Likewise, substandard management of foetal distress implied patients’ management intervention after diagnosis of foetal distress that did not fulfil all agreed criteria for management of foetal distress.

#### Compare prevailing practice with the agreed standard practice (third step)

The baseline of prevailing practice was compared with the standards of diagnosis and management of foetal distress and summarised. This implied that With reference to the agreed standard diagnosis and management of foetal distress in the audit procedure section, substandard diagnosis is a physician documented diagnosis of foetal distress that did not fulfil at least one major and one more criteria. Likewise for management, the substandard management of foetal distress implied patients’ management intervention after diagnosis of foetal distress that did not fulfil all agreed criteria for management of foetal distress. A team of three evaluators, including one consultant, one specialist and one nurse midwife, were trained to assess the fulfilment of the criteria, and so evaluated the recorded practice against the agreed standard criteria. In case of disagreement, the decision was based on panel majority.

#### Feedback and decision on intervention to improve practice (fourth step)

The summary of the analysed fulfilled standards of diagnosis and management of foetal distress were presented and discussed at the 1-day workshop by 65 stakeholders in 7 groups of 8–10 people, including obstetricians, obstetric residents and registrars, nurse midwives, maternity ward attendants, anaesthetists, pharmacists and laboratory technicians from maternity wards at MNH and Dar es Salaam, and Pwani public health facilities. From this feedback workshop, changes in practice were recommended and a summary of each discussion group’s recommended interventions were presented to all the stakeholders for authentication (Tables 3 and 4).

**Table 3:** Summary of recommended interventions to improve diagnosis of foetal destress following baseline audit feedback

1. Posting the criteria of standard diagnosis in the labour ward and operating theatre
2. Regular reminder of the use of the diagnostic criteria during grand rounds and routine work
3. Confirm the diagnosis of foetal distress using the posted criteria before taking the patient to or receiving the patient in the operating theatre
4. Provide Fetal Dopplers and train doctors and midwives how to use a Fetal Doppler and interpret foetal heart rate and rhythm
5. Doctors at the referring points should use the diagnostic criteria to ascertain the diagnosis before making referrals at MNH

**Table 4:** Summary of recommended interventions to improve management of foetal distress following baseline audit feedback

I. Interventions to improve pre-operative assessment and management of foetal distress
1. Specialist on call should stay within the hospital compound at all times
2. In case of emergency, midwives should communicate directly with the specialist when residents on call are unavailable
3. Specialist on call should make regular visits in the labour ward, preferably during morning major ward round and afternoon and evening service ward rounds
4. Strengthen documentation during patient review, either by self, over the phone, or during major ward round
5. Provide Fetal Dopplers and vacuum extractors, and re-train doctors and midwives on foetal heart monitoring and vacuum extraction
6. Doctors should register their private mobile phone numbers in the doctors’ free call system provided by Voda Com mobile company to improve communications and consultations within MNH and with external referring points
II. Interventions to reduce decision to delivery interval
1. In cooperation with and appraisal of ‘the Golden hour’ of decision to delivery intervention as part of the “Kaizen” hospital quality improvement system
2. Enforce mandatory prior communication of foetal distress to operating theatre after decision of CS to insist on the level of emergency and facilitate prioritisation in theatre
3. Re-organise midwives’ shifts to cater for increased workload during off hours and public holidays
4. Strengthen leadership and re-organise feedback meetings and clinical rounds to encourage teamwork and constructive routine perinatal audits among doctors and midwives
5. Care providers in theatre including obstetrician/resident on call, theatre nurse and anaesthesiologists/anaesthetists, should triage patients together in the pre-operative ward
6. Provide extra operating space by opening the gynaecology theatre for obstetric patients in the event of being overwhelmed by the workload in the two obstetric theatres
7. Referred patients should be sent to MNH when the decision of referral is made, rather than accumulating several patients to be referred all at once

#### Implementation of the recommended interventions (fifth step)

The summary of recommended interventions was presented to all the care providers at the labour ward, obstetric theatre and to the hospital administration. Representatives from the five referring hospitals also conducted a meeting to discuss the agreed interventions to improve diagnosis and management of foetal distress at the referring point. The interventions were then implemented for 4 months (March to June, 2015).

#### Re-evaluation of practice (Sixth step)

During the re-audit, the same data that were gathered at baseline were collected to facilitate evaluation of the intervention.

### Main outcome measures

The main outcome measure was fulfilment of at least one major and one additional minor criterion for standard diagnosis, and fulfilment of all nine criteria for the standard management of foetal distress.

### Sampling and statistical analyses

The sample size was calculated using Epi info 7. In 2012, 13 % of CSs were performed due to foetal distress and out of the 10,433 deliveries, 49.3 % were CSs. Therefore, the number of women with foetal distress was 669 (MNH obstetric database, unpublished report). Therefore, for the baseline audit, a minimum of 248 patients was required, assuming that the proportion of substandard care in diagnosis and management was unknown, and so estimated at 50 % with an absolute precision of 5 %. Given that half of the incidents of foetal distress received substandard care (51.6 %) in the baseline audit, in order to detect a 10 % improvement of care, we needed 250 parturients for the re-audit. Eligible participants were recruited every morning at 08.00 h from patients that delivered the previous night, and every afternoon at 16.00 h for those that delivered during the day hours of the same day. Participants were consecutively recruited until the required sample size was reached.

Data were entered and analysed using SPSS. A difference in percentage fulfilment of ≥1 for major and ≥1 for minor criteria for diagnosis, and all nine criteria for management, and percentage of fulfilment of each major and minor criterion for diagnosis and management at the baseline and re-audit were analysed using Student’s *t*-test. For each criterion, missing data were classified as ‘criteria not fulfilled’. The difference in median time between: a) decision and delivery by CS, b) decision and theatre arrival, and c) theatre and delivery at baseline and re-audit, was analysed using median test. Differences in antenatal characteristics and obstetric history between baseline and re-audit for substandard diagnosis and management were analysed using Chi-square test or Fisher’s exact test, when appropriate. The level of significance (α) was set at *p* < 0.05.

## Results

There were 248 patients with incidents of foetal distress in the baseline audit and 251 in the re-audit. There was a significant improvement in diagnosis of foetal distress between the baseline audit and the re-audit (52 % vs. 68 %; *p* < 0.001), partly due to a significant increase in the re-assessment of abnormal foetal heart rate and foetal heartbeat after immediate care (32 % vs. 50 %; *p* < 0.001) (Table 5).

**Table 5:** Percentage of fulfilled criteria for diagnosis between baseline and re-audit

Standards	Percentage of fulfilled criteria in diagnosis				
	Baseline audit (*n* = 248)		Re audit (*n* = 251)		*p*-value
	M	%	M	%	
Fulfilled ≥1 major and ≥1 minor	0	51.6	0	68.1	<0.001
Fulfilled major criteria					
Recorded abnormal FHR* <100/>180	11	34.7	33	36.7	0.65
Recorded irregular FHBs*	25	61.7	38	68.5	0.11
Fulfilled minor criteria					
Recorded reduced foetal movements	0	32.7	0	31.5	0.78
Recorded presence of fresh meconium-stained liquor	90	35.4	20	31.5	0.34
Re assessment of abnormal FHB* after immediate care	122	31.5	43	50.2	<0.001

Student’s *t*-test for test of difference between baseline and re-audit (M = Missing)

*FHR Foetal heart rate, *FHBs* Foetal heart beats, *FHB* Foetal heart beat

There was a significant improvement in the management of foetal distress between the baseline and the re-audit (0.8 % vs.8.8 %; *p* < 0.001) due to an improvement of change in maternal position (38 % vs. 87 %; *p* < 0.001) and fulfilled timeline of CS intervention, both in the duration of time between decision to arrival at theatre (23 % vs. 38 %; *p* = 0.001) and in the duration of time between arrival at theatre to delivery (15 % vs. 25 %; *p* = 0.006) (Table 6). The latter was confirmed by the analysis of median time from decision of CS to theatre (60 vs. 40 min; *p* = 0.002) and from theatre to delivery (60 vs. 51 min; *p* = 0.020) (Table 7).

**Table 6:** Percentage of fulfilled standards for management of foetal distress at baseline and re-audit

Standards	Percentage of fulfilled criteria in management				
	Baseline audit (*n* = 248)		Re-audit (*n* = 251)		*p*-value
	M	%	M	%	
Fulfilled all (9) criteria for management	0	0.8	0	8.8	<0.001
Fulfilled criteria for immediate care					
Intravenous rehydration	4	95.6	0	95.2	0.85
Change of maternal position	0	37.9	0	86.5	<0.001
Review by a senior	0	35.9	0	44.2	0.057
Fulfilled pre-operative care					
Urethral catheterization	0	95.6	0	98.8	0.053
Blood grouping and cross matching	0	97.2	0	98.8	0.22
Administration of pre-operative prophylactic antibiotic	0	95.6	0	95.6	0.97
Informed consent	0	96.4	0	98.8	0.87
Presence of pre-operative check list	0	98.0	0	98.8	0.50
Decision to delivery interval	0	10.1	0	22.3	<0.001
^a^Fulfilled timeline of CS intervention					
Decision to delivery time (≤60 min)	0	9.3	7	20.5	0.001
Decision to theatre time (≤30 min)	0	23.3	0	37.9	0.001
Theatre to delivery time (≤30 min)	0	15.0	0	25.4	0.006

Student’s *t*-test for test of difference between baseline and re-audit (M = Missing)

^a^denotes cases of women delivered by CS only

**Table 7:** Median and range of time interval of intervention in baseline and re-audit

Timeline of intervention	Median and range of time interval (minutes)		*p*-value
	Baseline audit	Re-audit	
From decision to delivery	125 (30–555)	100 (28–472)	<0.001
From decision to theatre	60 (10–440)	40 (7–230)	0.002
From theatre to delivery	60 (15–480)	51 (10–316)	0.020

Median test for test of difference between baseline and re-audit in women delivered by CS

The effect of the intervention by obstetric history and patient category factors is presented in Table 8. The significant improvement in substandard diagnosis was for patients who were 20 years older, primiparas, term deliveries and both public referred and private non-referred patients (all *p* ≤ 0.01). In substandard management, significant improvement was seen in women aged 20–34 years, primiparas and parity between 2 and 4, term deliveries, and both public referred and private non-referred patients.

**Table 8:** Percentage of substandard diagnosis and management during baseline (*n* = 248) and re-audit (*n* = 251) by obstetric history and patient category

Characteristic	Substandard diagnosis			Substandard management		
	Baseline audit	Re-audit	*p*-value	Baseline audit	Re-audit	*p*-value
	(%)	(%)		(%)	(%)	
Maternal age (years)						
< 20	57.9	26.7	0.07	100	100	na*
20–34	46.4	34.3	0.01	98.9	90.2	<0.001
≥ 35	54.6	18.8	0.003	97	100	1
Parity						
1	54.4	28.9	<0.001	99.0	89.8	0.004
2–4	45.3	35.3	0.11	99.3	93.9	0.02
≥ 5	25.0	28.6	0.88	100	71.4	0.20
Gestational age (weeks)						
< 37	50.0	0	na*	100	0	na*
37–42	48.6	32.0	<0.001	99.2	91.2	<0.001
>﻿ 42	33.3	0	1	100	100	na*
Referral						
Yes	47.5	38.4	0.10	99.3	91.8	0.002
No	37.7	20.6	0.009	99.1	90.2	0.005
Payment category						
Public	51.3	37.5	0.01	99.4	91.3	0.001
Private	43.3	22.0	0.002	98.9	89.0	0.005
Referral and payment category						
Referred public	53.8	38.2	0.008	99.2	92.4	0.005
Non-referred public patient	38.5	0	0.53	100	100	na*
Referred private	40.0	50.0	1	100	50.0	0.18
Non-referred private patient	43.8	21.4	0.002	98.8	89.9	0.015

Chi-square test or Fisher’s exact test for test of difference between first and re-audit

*na not applicable

## Discussion

Criteria-based audit resulted in significant improvement in the diagnosis and management of foetal distress by using available resources. Following 4 months of implementing the care providers’ suggested interventions, standards of diagnosis increased by 30 % while that of management increased tenfold. The main contributors to improved standards of diagnosis included re-assessment of the rate and rhythm of foetal heartbeat after hydration and change of maternal position, which could be a result of the introduction of “Moyo” Fetal Dopplers [29]. Improved care included upgrading of clinical acumen in detecting and combining neurological and cardiovascular signs of foetal distress [30–32], which formed the basis for our criteria for diagnosis. Immediate care after diagnosis by changing of the maternal position, and reducing the duration of time from decision to delivery, reduced the percentage with substandard management. Our audit interventions also improved care among women who were referred, especially for the public non-paying women who suffer from delays in care in the referral system, and hence are subject to severe morbidity of both mother and foetus [33, 34].

Pinard stethoscopes and Fetal Dopplers are locally available and affordable instruments that could be competently used by the care providers. Fetal Dopplers were used to differentiate between maternal and foetal heartbeats, and also to confirm abnormal FHR, even by other care providers in the room [9]. Uniquely in our case, “Moyo” Fetal Dopplers had an advantage of 30 min’ tracing of foetal heart activity [35]. Similar to other Fetal Dopplers, the “Moyo” Doppler was also regarded as a confirmatory test to clear or confirm suspected abnormal FHR raised by Pinard auscultation. Our study group included low-risk pregnancies and so intermittent monitoring was performed using both methods; and hence, further ensured frequent contacts with healthcare providers, the opportunity for social and clinical support, and direct palpation of foetal movements and maternal contractions [36].

East and co-workers [14] showed that the presence of abnormal foetal heartbeat increases the likelihood of foetal distress, but did not solely confirm foetal distress, or a need for CS. On the other hand, equivocal foetal heartbeats may not exclude the diagnosis of foetal distress [37]. Thus, early detection and adequate management of foetal distress can only be achieved through improved skills of care providers and adherence to clear, accessible and evidence-based standard management guidelines within the existing health system. Our agreed standards were in line with the WHO [26] and FIGO guidelines [9] of intrapartum foetal heart monitoring by defining the cut-off point for foetal bradycardia as ≤100beats/min. This cut-off point was also meant to avoid confusion between foetal bradycardia and maternal heart rate. Similarly, the definition of foetal tachycardia was ascertained at FHR >180beats/min by both Pinard auscultation and Fetal Doppler [38, 39]. Pinard auscultation demanded skills and experience in identifying abnormal deceleration including late and prolonged deceleration of foetal distress [9]. Therefore, it was important to operationalise the criteria by defining abnormal foetal heart rhythms that specifically associate with persistent non-uniform foetal heart beats in between uterine contractions [26]; and hence, avoid inclusion of early deceleration as criteria for suspected foetal distress.

Unlike previous reports [5, 40], in this study, reduced foetal movements (RFM) were not solely considered to be indicative of foetal distress, but rather, as a sign of placental or umbilical cord dysfunction [41–43]. Thus, additional signs of variability of foetal heart rate and/or rhythm were prerequisites of diagnosis. Supported by the findings of other studies [44, 45], we regarded RFM as a minor criterion to indicate foetal compromise, in view of the subjectivity of the perception of foetal movement in during labour [46, 47], especially in woman with a prior history of adverse outcome, and depending on foetal position [48]. Cessation of foetal movement was used as a minor criterion, to indicate impending foetal death, while gradual diminishment of foetal activity can indicate chronic foetal compromise. Therefore, RFM in the presence of abnormal foetal heart rate or rhythm was an acceptable sign of foetal distress [49].

Meconium-stained liquor could not be solely considered foetal distress, unless combined with foetal heartbeat abnormality [50]. Meconium-stained liquor could be a physiological response of a mature foetal gastrointestinal tract or relaxation of the anal sphincter in response to foetal hypoxia [30, 31]. van Boagert and co-workers, in their audit of decision to conduct CS due to non-reassuring foetal beats and/or meconium-stained liquor, found no difference in neonatal outcome between the CS group and the group who had vaginal deliveries with meconium-stained liquor [51]. Therefore, the acceptable diagnosis of foetal distress was when meconium-stained liquor was associated with foetal heartbeat abnormality.

The improved standards of management in each case required 100 % fulfilment of agreed standards because our criteria were similar to those defined in the national guideline. The adoption of national or international definitions does not ensure the effectiveness of CBA in low-resource settings. This could be a result of lack of feasibility of case selection, failure of agreement of the criteria of standard practice, and unavailability and inaccessibility of adequate documentation. Furthermore, success of audit requires assurance that feedback will be helpful in improving the quality of care. The criteria used in our case were realistic because the national referral hospital was able to provide comprehensive emergency obstetric care (EmOC), as recommended by the WHO.

Despite finding a significant reduction of time between the decision and the delivery, structural limitations of operating space could have hindered further shortening of the duration of the pre-operative interventions. The recommended use of the gynaecology theatres was designed to decongest heavy use of the obstetric theatre during busy periods. However, the process of transferring patients to the gynaecology theatre imposed extra work on care providers, which including wheeling patients along a corridor three times longer than that from the labour room to the obstetric theatre, carrying CS kits, and the movement of extra staff to receive and resuscitate the new-born. Thus, this intervention might have compromised care providers’ willingness and readiness for change [22].

The apparent lack of literature on CBAs of foetal distress marks the key contribution of this CBA in research and clinical management, particularly in low resource settings. Unavailability of standard tests for foetal distress (CTG and blood gas/lactate testing), at MNH and other low resource settings, facilitated the application the agreed criteria of standard diagnosis as local clinical management guideline, which was absent. In keeping with the evolving evidence-based practice, adaption of the agreed standards of care may require further upgrading of criteria for diagnosis and management of foetal distress in future audits. Effectiveness of audit-feedback intervention to reduce unnecessary CS can be further improved by investing in health resources, including CTG and blood gas/lactate analysis.

Routine use of high-technology diagnostic techniques, such as CTG and blood gas/lactate analysis, has not demonstrated a positive impact in preventing severe perinatal morbidity [52]. On the other hand, despite the limited availability of equipment to conduct foetal blood gas/lactate analysis in Tanzania, the risks associated with foetal scalp blood sampling in an area with high HIV prevalence among pregnant women (~7 %) [53], could discourage foetal blood gas/lactate analysis as a routine test. Thus, our findings challenge the healthcare system to motivate and improve care providers’ skills, and to upgrade pregnancy and delivery surveillance capability by introducing Fetal Dopplers and lactate test at all delivery facilities, and CTG and blood gas analysis equipment in referral facilities; so as to increase the facilities’ ability to accurately detect foetal distress.

The strength of this study includes the use of a piloted audit form that improved the relevance of selected criteria, and hence, increased criteria validity. Furthermore, the stepwise revision of the audit form during piloting increased clarity of the questions as well as the reliability of results. Training and monitoring of data collectors, regular accounting of missing cases, and evaluation and filling of the missing data, also improved validity of results, and subsequently, reliability of the results [54]. However, this study also had limitations. Care providers’ consensual list of criteria and interventions originated from WHO manuals, FIGO guidelines of intrapartum monitoring and national guidelines that went through multiple levels of revisions and adjustments to reach consensus to guarantee feasibility criteria and interventions. As a result, the universal validity of the criteria was reduced. This audit assessed the quality of care from data in the hospital case files which may not have provided sufficient information about the real situation in the delivery room and theatre in terms of the number of available staff per patient. Finally, although there was no difference in the basic characteristics of the study population between baseline and re-audit, the differences in the general condition of patients, such as level of exhaustion and degree of hydration, were not assessed.

Quality of care improvements are expected to reduce adverse maternal and new-born outcomes. Analysis of outcome indicators promises a more comprehensive picture of the impact of audit. Continued medical education, and monitoring and updating of clinical guidelines should also be a priority for providing sustainable evidence-based care to mothers and their new-borns [55].

## Conclusion

This criteria-based audit was able to detect substandard diagnosis and management of foetal distress, leading to improved care by using feedback and available resources. Because timely access to good quality care is a professional responsibility and every patient’s right, further investment in EmOC, even in low-resource settings, should be a priority.

## Abbreviations

CBA: Criteria based audit
CS: Caesarean section
CTG: Cardiotocography
EmOC: Emergency obstetric care
FHB: Foetal heart beats
FHR: Foetal heart rate
FIGO: International Federation of Obstetricians and Gynaecologists
MNH: Muhimbili National Hospital
RFM: Reduced foetal movements
WHO: World Health Organisation
